# Identification and Characterization of ABA Receptors in *Oryza sativa*

**DOI:** 10.1371/journal.pone.0095246

**Published:** 2014-04-17

**Authors:** Yuan He, Qi Hao, Wenqi Li, Chuangye Yan, Nieng Yan, Ping Yin

**Affiliations:** 1 State Key Laboratory of Bio-membrane and Membrane Biotechnology, Beijing, China; 2 Center for Structural Biology, School of Medicine, Beijing, China; 3 School of Life Sciences, Tsinghua University, Beijing, China; 4 National Key Laboratory of Crop Genetic Improvement and National Centre of Plant Gene Research, Wuhan, China; 5 College of Life Sciences and Technology, Huazhong Agricultural University, Wuhan, China; Van Andel Research Institute, United States of America

## Abstract

Abscisic acid (ABA) is an essential phytohormone that regulates plant stress responses. ABA receptors in *Arabidopsis thaliana* (AtPYLs) have been extensively investigated by structural, biochemical, and *in vivo* studies. In contrast, relatively little is known about the ABA signal transduction cascade in rice. Besides, the diversities of AtPYLs manifest that the information accumulated in Arabidopsis cannot be simply adapted to rice. Thus, studies on rice ABA receptors are compulsory. By taking a bioinformatic approach, we identified twelve ABA receptor orthologs in *Oryza sativa* (japonica cultivar-group) (OsPYLs), named OsPYL1–12. We have successfully expressed and purified OsPYL1–3, 6 and 10–12 to homogeneity, tested the inhibitory effects on PP2C in *Oryza sativa* (OsPP2C), and measured their oligomerization states. OsPYL1–3 mainly exhibit as dimers and require ABA to inhibit PP2C’s activity. On the contrary, OsPYL6 retains in the monomer-dimer equilibrium state and OsPYL10–11 largely exist as monomers, and they all display an ABA-independent phosphatase inhibition manner. Interestingly, although OsPYL12 seems to be a dimer, it abrogates the phosphatase activity of PP2Cs in the absence of ABA. Toward a further understanding of OsPYLs on the ABA binding and PP2C inhibition, we determined the crystal structure of ABA-OsPYL2-OsPP2C06 complex. The bioinformatic, biochemical and structural analysis of ABA receptors in rice provide important foundations for designing rational ABA-analogues and breeding the stress-resistant rice for commercial agriculture.

## Introduction

Abscisic acid (ABA) is an essential phytohormone that not only regulates seeds dormancy, germination and seedling growth [1], [2], but also is involved in responses to environmental stresses such as drought, high salinity and chilling [3], [4]. *In vitro* reconstitution in Arabidopsis assays defined three core components within the minimal ABA signaling pathway: ABA receptor PYR1/PYL/RCAR protein family (hereafter referred to as AtPYLs for simplicity), the negative regulator type 2C protein phosphatase (short for PP2Cs) and the positive regulator Class III SNF1-related protein kinase 2 (SnRK2s) [5]. There are 14 members of the AtPYLs that are highly conserved in amino acid sequences and share three-dimensional structural similarities [6], [7], [8]. AtPYLs have an ABA-binding pocket whose entrance is surrounded by conserved CL1–CL4 loops. In the presence of ABA, the CL2 loop undergoes conformational changes and mediates the interaction with PP2C, resulting in the inhibition of the phosphatase activity of PP2Cs, and consequently leads to the activation of the kinases SnRK2s to turn on downstream gene expression [9], [10].

Most of the ABA receptors studies were carried out on Arabidopsis, however, less is known about the ABA signal transduction cascade in agricultural crops, especially in rice, the widely consumed staple crop by the largest human population [11]. The rice ortholog of the ABA receptor in *Oryza sativa* cv Dong-Jin, OsPYL/RCAR5, was recently identified as a positive regulator in seed germination and early seedling growth *in vivo* [12], [13]. Nevertheless, over a prolonged duration of time, the lack of biochemical and structural information of ABA receptors impedes the biology development on rice. Moreover, not to mention that of rice, in Arabidopsis, the high sequence similarities among 14 AtPYL receptor members cannot simply explain their diverse biochemical properties. For instance, AtPYR1, AtPYL1–2 are constitutive homodimers in solution and require ABA to inhibit PP2C’s activity, whereas some AtPYLs, exemplified by AtPYL10, exhibit monomeric forms and inhibit PP2Cs in an ABA-independent manner [14]. A single amino acid variation on AtPYL2 was found to determine the perception of pyrabactin, an agonist of ABA [15], [16], [17]. Particularly, AtPYL13 is unresponsive to ABA and selectively inhibits phosphatase activity of PP2CA [18], [19]. These findings implicated, despite high sequence homology, the biochemical properties of ABA receptors from Arabidopsis or even from distinct plant species can be greatly disparate.

**Figure 1:**
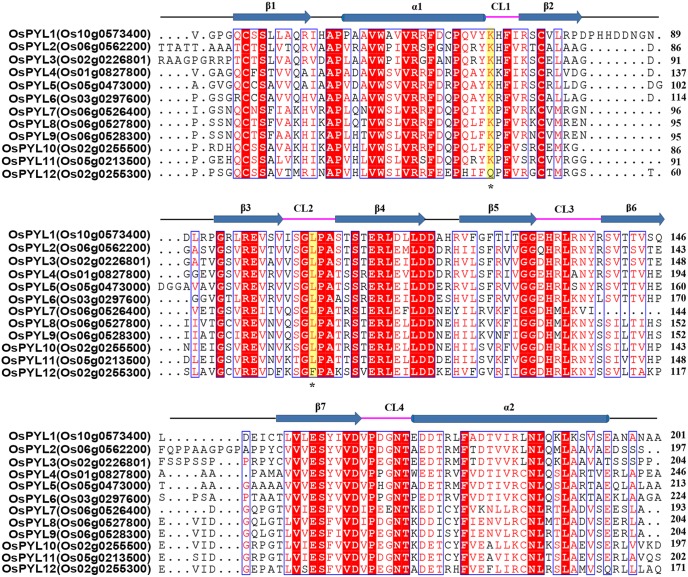
Sequence alignment of the twelve characterized OsPYLs. Secondary structural elements are indicated above the primary sequence. Helices and strands are showed as blue cylinders and arrows, respectively. The four conserved loops CL1–CL4 are highlighted by magenta lines. The key residue of OsPYL12 which are different from other OsPYLs are marked with star and colored yellow.

Therefore, extensive characterizations on rice ABA receptors are necessary for their scarce information and property diversities. We employed computational comparative genomics search for the identification of ABA receptors in rice, and retrieved twelve rice orthologs in *Oryza sativa* (japonica cultivar-group) (hereafter referred to as OsPYLs for simplicity) (Figure 1). We sought to express these OsPYLs proteins in *E.coli*. The recombinant proteins showed different PP2C inhibition manners and existed in various oligomerization states. These diversities urge us to pursue detailed structural information of OsPYLs. Eventually, we determined the crystal structure of ABA-OsPYL2-OsPP2C06 ternary complex. The first structure of ABA receptor in rice revealed the molecular mechanism of ABA sensitivity and phosphatase inhibition of OsPYLs. Along with bioinformatic, biochemical and structural information, our study in rice ABA receptors provides useful information for ABA-analogues design and trans-genetic rice development.

## Materials and Methods

### Protein Preparation and Crystallization

OsPYLs homologues and OsPP2Cs were subcloned from the *Oryza sativa* cDNA library using standard PCR-based protocol. All proteins were purified according to the protocol described before [9]. All the OsPYLs and OsPP2Cs proteins were expressed in *E.coli* strain BL21 (DE3) using vector pET-15b induced at 22°C for 14 hours. To obtain a stable ABA-OsPYL2-OsPP2C06 complex, the His6-tagged core domain (residues 132–467) of OsPP2C06 was coexpressed with OsPYL2 in *E.coli* BL21 (DE3). Before IPTG induction, 4 mM MnCl_2_ were added to the culture medium. After induction at 22°C for 14 h, cells were collected and lysed in a buffer containing 25 mM Tris, pH 8.0, 150 mM NaCl. For complex purification, additional 0.2 mM (+)-ABA (Jingkehongda Ltd.) was added. The proteins were purified with Ni-NTA resin (Qiagen), followed by anion exchange chromatography (Source-15Q, GE Healthcare) and size exclusion chromatography (Superdex-200, GE Healthcare) in the same buffer. All the protein for assay were exchanged to buffer containing 25 mM HEPES (pH 7.0), 150 mM NaCl. Before crystallization, 0.2 mM (+)-ABA (Sigma-Aldrich) was mixed with 0.1 mM protein complex. Crystals were grown in the well buffer contained 0.1 M Bis-Tris, pH 5.5, 0.2 M MnCl_2_ and 20% PEG3350. Crystals grew to full size after a week.

### Data Collection, Structure Determination, and Refinement

Data of the ABA-OsPYL2-OsPP2C06 was collected at the Shanghai Synchrotron Radiation Facility (SSRF) beamline BL17U and processed with the HKL2000 package [20]. Further processing was carried out using programs from the CCP4 suite [21]. Data collection statistics are summarized in Table 1. The ABA-OsPYL2-OsPP2C06 complex structure was solved by molecular replacement (MR) with AtABI1 (3KDJ) and AtPYL2 (PDB code: 3KDH) as search ensembles using the program PHASER [22]. Model building and refinement were performed with COOT [23] and PHENIX [24].The atomic coordinated and structure factors of ABA-OsPYL2-OsPP2C06 have been deposited in Protein Data Bank with accession number 4OIC.

**Table 1:** Data collection and refinement statistics.

	ABA/OsPYL2/OsPP2C06
**Data collection**	
Space Group	C2
Cell dimensions	
* a, b, c (*Å)	164.19, 43.52, 73.99
α, β, γ (°)	90, 92.53, 90
Wavelength (Å)	0.9795
Resolution (Å)	50∼2.00
*R_merge_* (%)	8.1 (32.7)
*I/*δ *I*	11.3 (2.1)
Completeness (%)	97.5 (88.4)
Number of measured reflections	77,990
Number of unique reflections	34,921
Redundancy	2.2
Wilson B factor (Å^2^)	19.7
Resolution (Å)	50∼2.00
No. reflections	34,912
*R*_work_/*R*_free_ (%)	16.99/20.12
No. atoms	
Protein	3,748
main chain	1,954
side chain	1,794
Ligand	19
Ion	6
Water	448
*B*-factors	
Protein	34.17
main chain	33.86
side chain	34.51
Ligand	23.34
Ion	24.90
Water	37.48
R.m.s. deviations	
Bond lengths (Å)	0.009
Bond angles (°)	1.257
Ramachandran plot statistics (%)	
Most favourable	90.3
Additionally allowed	9.7
Generously allowed	0.0
Disallowed	0.0

Values in parentheses are for the highest resolution shell. *R_merge_* = Σ_h_Σ_i_|*I_h,i_*-*I_h_*|/Σ_h_Σ_i_ *I_h,i_*, where *I_h_* is the mean intensity of the *i* observations of symmetry related reflections of *h*. *R* = Σ|*F_obs_*-*F_calc_*|/Σ*F_obs_*, where *F_calc_* is the calculated protein structure factor from the atomic model (R_free_ was calculated with 5% of the reflections selected).

### Isothermal Titration Calorimetry (ITC) Assays

The ITC experiments were performed with VP-ITC Microcalorimeter (MicroCal) as described previously [9]. The stoichiometry between ligand and protein was set to one for all analyses.

### Phosphatase Activity Assay

The protocol of phosphatase activity assay was performed as described previously [9]. Each reaction was performed in 50 µL reaction system. The concentration of phosphatase (OsPP2C06 3 µM, OsPP2C09 1.8 µM) were adjusted to the linear range of absorbance measurement. The concentrations of OsPYLs were adjusted to match the molar ratios as indicated in this manuscript. When ABA was required, its concentration was ten fold of OsPYLs. Data were measured at the wavelength of 630 nm. The readout of the reaction without phosphatase was subtracted from all the assay data. Data are means ±SD from three different experiments.

### Size-Exclusion Chromatography

The Size-Exclusion Chromatography (SEC) were performed with a SD200 (Superdex-200 HR10/300 GL, GE Healthcare) in the buffer containing 25 mM HEPES (pH 7.0), 150 mM NaCl. For examination of the oligomerization state of OsPYLs, 500 µL OsPYLs (OD_280_ = 0.75) were applied.

### Static Light Scattering Measurements

Static light scattering (SLS) were performed on a DAWN HELLOS II instrument (Wyatt Technology, Santa Barbara, CA) at 18°C. The concentration of all the OsPYLs were about 2 mg/mL. Before the assays, light scattering detector was calibrated with albumin monomer standard. The data was treated by ASTRA software (Wyatt Technology, version 6.1).

### Analytical Ultracentrifugation Analyses (Au)

Sedimentation velocity was performed with analytical ultracentrifuge (Beckman Coulter) at 4°C. All OsPYL proteins (OD_280_ = 0.75) were applied at the speed of 40,000 rpm. Absorbance scans were obtained at 280 nm at the intervals of 0.003 cm size in a radical direction. The data was analyzed by ProteomeLab software (Beckman Coulter).

## Results

### Twelve Members of ABA Receptors in *Oryza Sativa*

To identify the PYL/RCAR orthologs from rice, 14 Arabidopsis PYL amino acid sequences were used as queries to search against the Rice Annotation Project Database (RAPDB) respectively [25], [26]. Sixteen candidates that showed e-values <e^−10^ in BLASTP analysis were found. In all reported structures of AtPYLs, several conserved key amino acids were found to be crucial for the function of ABA receptors. For example, the lysine located in CL1 loop contributes to the interaction with ABA, and a highly conserved CL2 loop mediates PYL-PP2C interaction. Thus, we omitted the proteins sequences that did not contain these key features in the searched results. By further confirming the gene and protein sequences on MSU Rice Genome Annotation Project (http://rice.plantbiology.msu.edu/) and NCBI (http://www.ncbi.nlm.nih.gov/) database, we finally selected twelve proteins named OsPYL1–12 respectively for following study (Figure 1). We named them roughly according to their sequence similarities with AtPYLs. The reported functional ABA receptor OsPYL/RCAR5 was corresponded to OsPYL11 in our nomenclature (Table S1 in File S1) [12]. These twelve OsPYLs exhibited very high sequence similarities. According to the sequence alignment with reported AtPYL1 and AtPYL2, they all contain four identical conserved loops which play important roles in ABA binding and PP2Cs interaction (Figure 1). Interestingly, in the primary sequence, OsPYL12 shares the highest similarity with AtPYL13, especially the key residues Gln48/Phe76 which correspond to Gln38/Phe71 in AtPYL13 (Figure 1) [19]. These two residues Gln38/Phe71 in AtPYL13 were predicted to deprive the ability of this protein on ABA binding [18], [19]. In fact, OsPYL12 is unable to bind to ABA via isothermal titration calorimetry (ITC) assay (unpublished data). These observations suggested that OsPYL12 is an ortholog of AtPYL13.

### Inhibition Effects of OsPYLs on PP2Cs Activity

To systematically investigate the functions of these OsPYL, we firstly examined the OsPYLs inhibitory effects on PP2Cs in the absence and presence of ABA. We tried to overexpress all of them in *E.coli*, and successfully purified OsPYL1–3, 6 and 10–12 to homogeneity (data not shown). Meanwhile, we cloned some of the reported Group A PP2Cs genes from *Oryza sativa* (japonica cultivar-group) cDNA library (short for OsPP2C) [27]. After expression of those cloned OsPP2Cs, some of them are prone to precipitate, while OsPP2C06 (Os01g0583100) and OsPP2C09 (Os01g0846300) had acceptable yield and decent behavior. OsPP2C06 has the highest sequence identities with ABI1, and OsPP2C09 is the closest relative to PP2CA in Arabidopsis which contains additional zinc finger domain. Thus, these two OsPP2Cs were chosen for further phosphatase inhibition study.

**Figure 2:**
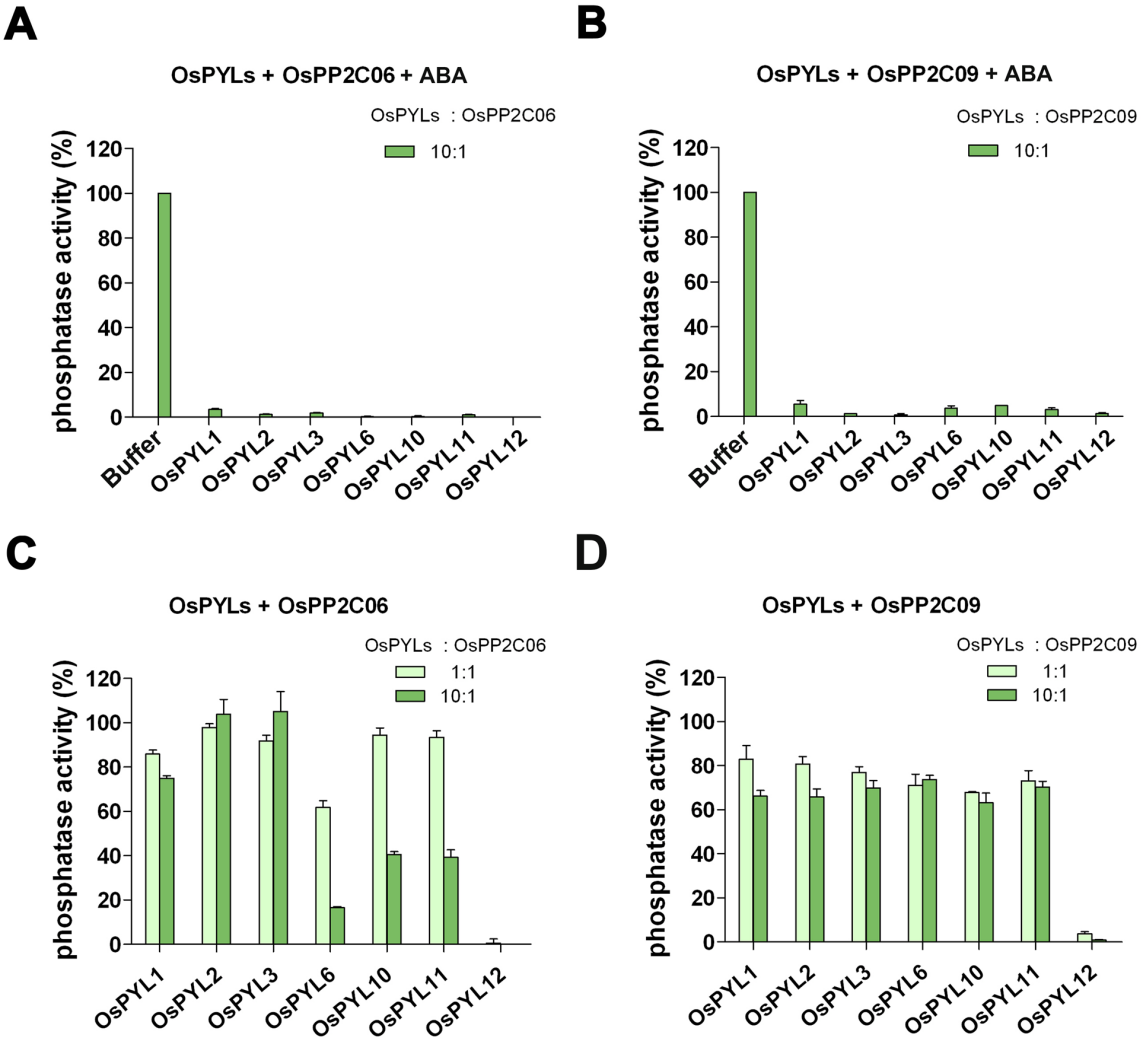
The inhibition effects of OsPYLs on OsPP2C06 and OsPP2C09. The inhibition effects on OsPP2C06 and OsPP2C09 in the presence of ABA were showed as panel A and panel B, and in the absence of ABA as panel C and panel D. The phosphatase activity was measured by the Ser/Thr phosphates assay system. The details of the experiments are described in the Material and Methods. The concentrations of each OsPYL were set with molar ratio to OsPP2Cs as 1∶1 and 10∶1 in the absence of ABA (colored light and dark green). Each reaction was repeated at least three times; error bars represent standard deviations.

The phosphatase activity was measured by the Ser/Thr phosphates assay system (see Materials and Methods). As expected, in the presence of ABA, all of the OsPYLs proteins could completely inhibit the phosphatase activity of OsPP2C06 and OsPP2C09 (Figure 2A, 2B), suggesting these OsPYLs are functional. We then examined the inhibitory effects on these two OsPP2Cs by OsPYLs in the absence of ABA, with the OsPYLs:OsPP2Cs molar ratios of 1∶1 and 10∶1. OsPYL1–3 failed to exhibit prominent inhibition effect on OsPP2C06. However, they exhibit weak inhibition effect on OsPP2C09. OsPYL6, 10, 11 were able to inhibit phosphatase activity of OsPP2C06 at the OsPYLs:OsPP2Cs molar ratio 10∶1 (Figure 2C). However, they failed to exhibit inhibition effect on OsPP2C09 (Figure 2D). Such observation is consistent with the inhibitory effect of AtPYLs on ABI1 and PP2CA [14]. Notably, OsPYL12 appeared to be a potent constitutive inhibitor of both these two OsPP2Cs. It almost abrogated the phosphatase activities of OsPP2C06 and OsPP2C09 at a OsPYLs:OsPP2Cs molar ratio 1∶1 (Figure 2C, 2D). Comparing to its ortholog AtPYL13 which specifically inhibit PP2CA independent of ABA, OsPYL12 is capable of suppressing the activity of either OsPP2C06 (orthologs of ABI1) or OsPP2C09 (orthologs of PP2CA) simultaneously. In summary, except for OsPYL12 as a constitutive inhibitor of PP2C, all the OsPYLs can inhibit PP2C’s activity in the presence of ABA, and in the absence of ABA OsPYL1–3 have no effect on PP2C yet OsPYL6, 10, 11 execute inhibitory function to various degrees. Thus, these OsPYLs could be categorized into ABA-dependent or ABA-independent subgroups according to their inhibition manners.

### Oligomerization States of Different OsPYLs

In previous study, ABA-dependent and ABA-independent inhibition manners of AtPYLs are related to their oligomerization states [14]. The dimeric AtPYLs require ABA to inhibit PP2C’s activity, but the monomeric AtPYLs are able to execute the PP2C inhibition function without ABA [14]. Is it possible that different phosphatase inhibition patterns by OsPYLs are intrinsically correlated with their distinct oligomerization states? Hence we sought to examine the oligomerization states of OsPYLs by size-exclusion chromatography (SEC), static light scattering (SLS) and analytic ultracentrifugation (AU).

**Figure 3:**
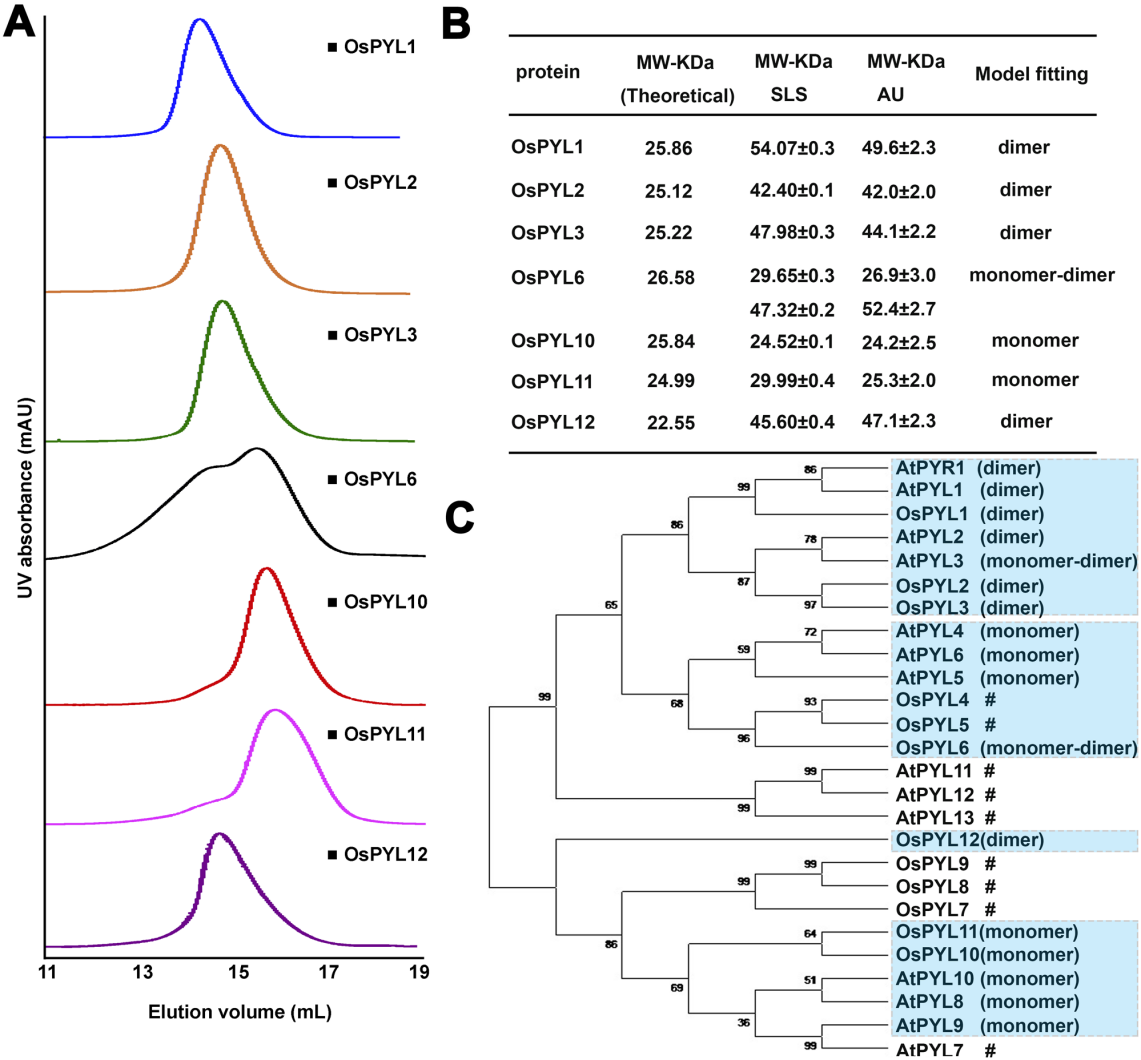
Oligomerization states of OsPYLs. (A) SEC analyses of OsPYLs. 500 µL OsPYLs (OD_280_ = 0.75) were applied to Superdex 200. (B) The molecular weight of OsPYL proteins were measured by static light scattering and analytical ultracentrifugation. (C) Phylogenetic tree of the 12 OsPYLs and 14 AtPYLs. Phylogenetic tree was constructed by neighbor-joining algorithms of MEGA 5.2. The labled # means the protein has no expression in *E.coli.*

The results demonstrated that OsPYL1–3 and OsPYL12 were eluted approximately at 14.8 ml in gel filtration (Figure 3A), which represented the molecular mass for two fold of their theoretical ones and agreed with the SLS and AU results (Figure 3B). OsPYL6, which had two peaks on gel filtration, may retain as an equilibrium state between a monomer and a dimer (Figure 3A). Further SLS and AU results also corroborated this conclusion (Figure 3B). OsPYL10 and OsPYL11, although they shared similar calculated molecular weights with OsPYL1–3 and OsPYL12, their elution volumes were approximately 1 ml after OsPYL1–3 and OsPYL12 in gel filtration and consistent with their molecular mass that of monomers (Figure 3A, 3B). In retrospect to the phosphatase inhibitory effects of OsPYLs, we found that the phosphatase inhibition effects are also correlated with the oligomerization states of OsPYLs, resembling those of AtPYLs. The dimeric OsPYL1–3 depend on ABA for PP2C inhibition. On the contrary, the monomeric-dimeric equilibrium state OsPYL6 and monomeric state OsPYL10, 11 inhibit PP2Cs in the absence of ABA. The only exception is OsPYL12, which existed as a dimer form yet constitutively inhibit PP2C without ABA.

In order to normalize the diversity of these OsPYLs, we generated a phylogenetic tree including all AtPYLs and OsPYLs proteins and classified them into four subgroups [28]. The molecular phylogenetic relations were consistent with protein oligomerization states: OsPY1–3 could be categorized into AtPYR1 and AtPYL1–3 dimeric group, OsPYL6 into AtPYL4–6 monomeric group, OsPYL10–11 into monomeric AtPYL8–10 group, and OsPYL12 exists as a dimer orphan (Figure 3C). All these results corroborate the previous conclusion that albeit ABA receptors share high sequence similarities, they are functionally diverse and exquisitely modulated.

### Crystal Structure of ABA-OsPYL2-OsPP2C06 Complex

**Figure 4:**
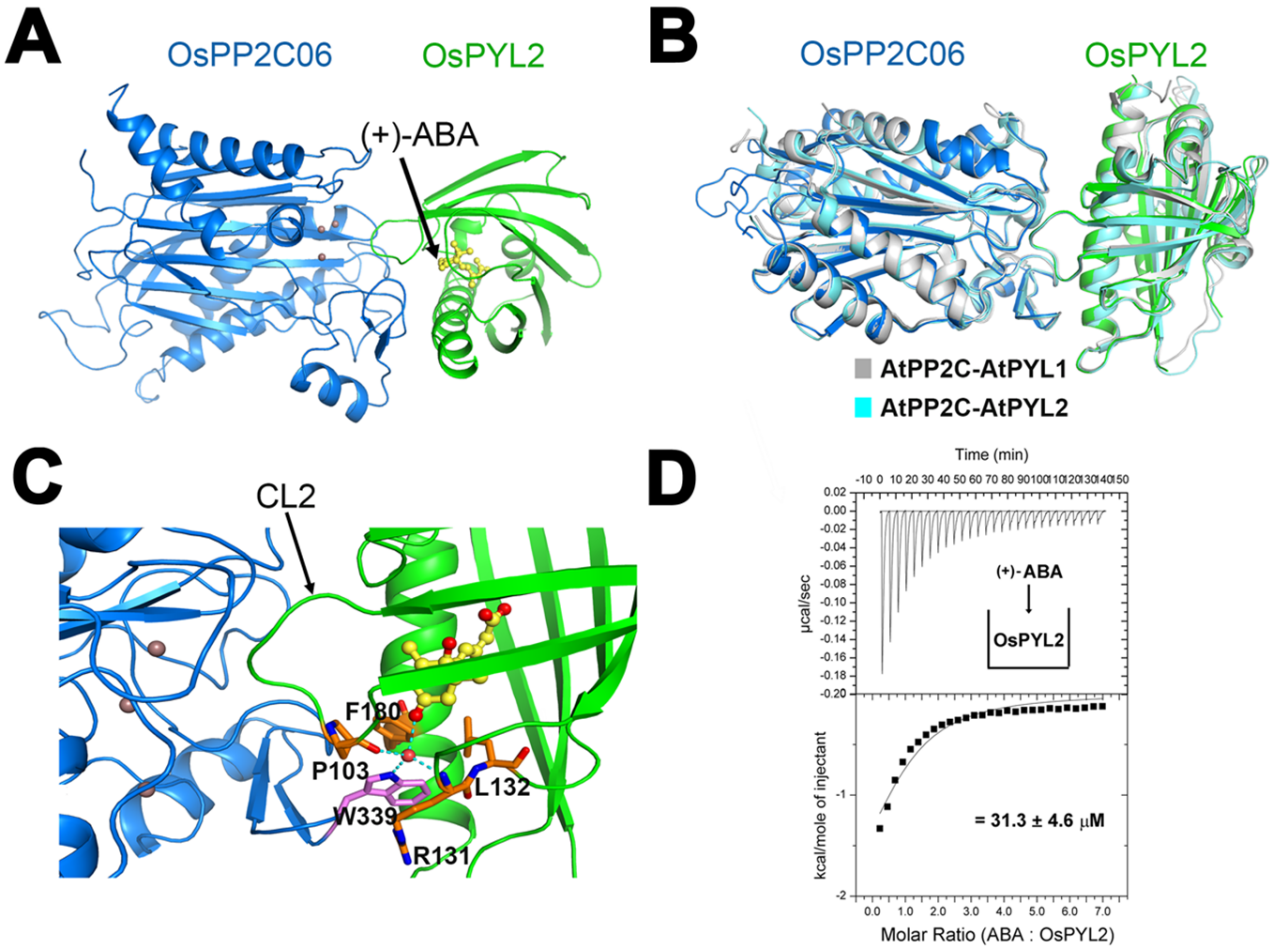
Structure of the (+)-ABA-OsPYL2-OsPP2C06 ternary complex. (A) Overall structure of the (+)-ABA-OsPYL2-OsPP2C06 ternary complex. Three purple spheres in the active site of OsPP2C indicate the three Mn^2+^ ions. (+)-ABA is shown in yellow ball-and-stick. (B) Superimposition of the structure of ABA-OsPYL2-OsPP2C06 ternary complex with ABA-AtPYL1-AtPP2C complex (PDB code: 3KDJ) and ABA-AtPYL2-AtPP2C (PDB code: 3UJL). Amino acids of ABA-AtPYL1-AtPP2C ternary complex are colored gray. Amino acids of ABA-AtPYL2-AtPP2C ternary complex are colored cyan. Amino acids of OsPP2C06 and OsPYL2 are colored blue and green, respectively. (C) The CL2 loop of OsPYL2 interacts with OsPP2C06 and partially occupies the catalytic site of OsPP2C06. Trp 339 of OsPP2C06 inserts into the hydrophobic pocket of OsPYL2 and interacts with the carbonyl oxygen of ABA through a water-mediated hydrogen bond. The red sphere indicates water molecular and the OH group in ABA. The side chain of Trp 339 is colored magenta. (D) Dissociation constant measurement between OsPYL2 and ABA binding by Isothermal Titration Calorimetry (ITC) assay.

These diversities of OsPYLs urged us to pursue for elucidating the ABA binding and PP2C inhibition mechanism of OsPYLs through structural study. In order to crystallize the complex, we co-expressed these OsPYLs with OsPP2C06 or OsPP2C09 for further structural study. After a myriad of crystal screening and optimization, we crystallized the complex of ABA-OsPYL2-OsPP2C06 in C2 space group and determined its structure by molecular replacement at 2 Å (Table 1) (Figure 4A) [9]. The overall structure of the ABA-OsPYL2-OsPP2C06 complex has no dramatic conformational change compared to that of ABA-AtPYL1-AtPP2C complex (PDB code: 3KDJ) with Root-Mean-Square deviation (RMSD) of 0.397 Å or ABA-AtPYL2-AtPP2C with the RMSD of 0.471 Å (PDB code: 3UJL) for all the Cα atoms (Figure 4B). The CL2 loop of OsPYL2 interacts with OsPP2C06 and partially occupies the catalytic site to block the substrate entrance (Figure 4C). This reveals the inhibition mechanism of OsPYL2 demonstrated in the phosphatases activity assay. OsPP2C06 adopts a typical PP2C fold which contains five-stranded antiparallel β-sheets sandwiched by two pairs of α-helices. Three Mn^2+^ ions are located at the catalytic site of OsPP2C06, which are usually observed in other PP2C structures. The side chain of Trp339 of OsPP2C06 inserts into the hydrophobic pocket (Leu132, Phe180) of OsPYL2 and forms a water-mediated hydrogen bond with the carbonyl oxygen of ABA (Figure 4C).

Similar to AtPYL2, OsPYL2 contains a typical START protein fold that comprised seven-stranded β-sheet and two α-helices, which creates a classical ABA-binding pocket. One ABA molecule is buried in the hydrophobic cavity of OsPYL2, unambiguously indicating that OsPYL2 can bind to ABA (Figure 4C). The acid head group of ABA form a charge-charge interaction with the side chain of K74, and also forms water-mediated hydrogen bonds with the surrounding residues E109, N188 and E162 (Figure S1 in File S1). These direct and water-mediated hydrogen bonds stabilize ABA in the hydrophobic cavity of OsPYL2, which are conserved with the mode of AtPYL2’s ABA perception [9], [10]. The hydrophobic pocket retains the similar size with AtPYL2 (Figure S2 in File S1). The amino acids in OsPYL2 consist of the narrow pocket (F76, V98, L102, P103, F180, V184) and the larger pocket (A104, S107, V125) are very conserved with AtPYL2 (Figure S2 in File S1) [9], [10]. These conserved amino acids point to the same directions with their corresponding residues in AtPYL2, thus render the size and shape of ABA binding pockets are almost identical between these two ABA receptors. We measured pocket volumes and areas by Chimera software [29]. The calculated volumes (OsPYL2: 900 Å^3^, AtPYL2: 934 Å^3^) and areas (OsPYL2: 655.3 Å^2^, AtPYL2: 666 Å^2^) also illustrate the pocket topology similarities. Isothermal titration calorimetry (ITC) assays were used to measure the binding affinity between OsPYL2 and ABA. ABA was titrated into protein, and a typical saturation curve was observed (Figure 4D). The dissociation constant (*K_d_*) of this binding was about 31.3 µM, consistent with the measured affinity of the Arabidopsis AtPYL2 ortholog (59.1 µM) [9]. Collectively, together with the specific hydrogen bonds and the size and shape of ABA binding pocket, this complex clearly demonstrated the ABA-binding capacity, specificity and PP2C inhibition mechanism of OsPYL2.

## Discussion

ABA is involved in many aspects of plant life cycle, such as seed germination, seedling development. Most studies were focused on ABA receptors of the model organism Arabidopsis, whereas only a few have been characterized in crops, especially in rice [11]. The fourteen AtPYLs have very high sequence identities but distinctive properties. Since the high similarity of sequence cannot explain the diversity of AtPYLs, whether the interpretation of ABA signaling cascade in Arabidopsis can be applied in rice is a remaining question.

In this study, we use computational genomics search to retrieve twelve rice ABA receptor orthologs in *Oryza sativa* (japonica cultivar-group), named OsPYL1–12. Among those which are able to be recombinant expressed in *E.coli,* OsPYL1–3 mainly stay as dimers and require ABA to inhibit PP2C’s activity. OsPYL6 exhibits in a monomer-dimer equilibrium state and is able to inhibit OsPP2C06 activity in the absence of ABA. OsPYL10, 11 largely exist as monomers and can inhibit OsPP2C06 phosphatase activity without ABA. Therefore, we can preliminarily categorize these OsPYLs into two subfamilies: the dimeric OsPYL1–3 which show ABA-dependent inhibition manners and the monomeric (or monomeric-dimeric) OsPYL6, 10, 11 which display ABA-independent inhibition patterns. This classification is agreed with that of AtPYLs [14]. Interestingly, OsPYL12 exists as a dimer. All the reported dimeric AtPYLs require ABA binding to induce conformational change, thus to inhibit PP2C’s activity. However, there is no obvious binding observed between OsPYL12 and ABA (unpublished data), which is in accordance with its ortholog AtPYL13. AtPYL13 was predicted to lose the ability of ABA binding on account of the unconserved residues Gln38/Phe71 that was corresponding to the Gln48/Phe76 in OsPYL12 (Figure 1). Moreover, AtPYL13 is reported to antagonize other AtPYLs independent of ABA [18], [19]. The interaction between AtPYL13 and other AtPYLs might reduce the binding affinity of other AtPYLs with ABA, therefore desensitizing their activities in response to ABA [19]. Since OsPYLs exhibit high sequence similarities and some of them can form homodimer, it is possible that some OsPYLs may form heterodimer as in Arabidopsis, especially OsPYL12 with other OsPYLs. Furthermore, the dimeric OsPYL12 largely functions as a constitutive inhibitor for PP2Cs, and it completely inhibits the phosphatase activities of OsPP2C06 and OsPP2C09 in the absence of ABA (Figure 3C, 3D). Thus, we suggest that OsPYL12 and AtPYL13 belong to a new family that different from other PYLs.

According to the structure of AtPYL13-PP2CA that AtPYL13 inhibits PP2CA’s activity in a monomer form [18] and the transgenic plants in which AtPYL13 is overexpressed exhibit accelerated water-use efficiency and enhanced drought resistance [19], we found that there are several pivotal yet unanswered questions still remain. How does the dimer form of OsPYL12 turns into monomer upon binding to PP2C? What is the difference between OsPYL12 dimer and OsPYL1–3 dimer? Besides, will OsPYL12 function the same way as AtPYL13 in accelerating adaptive responses to stress in rice? As long as the AtPYL13 cannot be solubilized and purified from *E.coli*, OsPYL12 represents a pseudo-ABA receptor for further study. Collectively, bioinformatic, biochemical and structural information of ABA receptor in rice provides practical information to the rational design of ABA analogues and the improvement of rice performance.

## Supporting Information

File S1 **Correspondence name nomenclatures and detailed structure descriptions.** Table S1. Correspondence relationships between two nomenclatures of ABA receptors in *Oryza sativa*. Figure S1. The 2Fo-Fc electron density map of (+)-ABA and the interactive residues in OsPYL2. (+)-ABA was shown as yellow sticks. The red spheres stand for water, the blue stick for nitrogen atom and the red stick for oxygen atom. Figure S2. The pocket residues surrounding around (+)-ABA in OsPYL2 (left, green) and AtPYL2 (right, cyan). The conserved surrounding amino acids consist of the similar pocket size and topology between these two ABA receptors. The conserved residues are colored brown and (+)-ABA is colored yellow.
(DOC)
