# Supplementary material for: Identification and Characterization of ABA Receptors in Oryza sativa
Source: PLoS One. 2014 Apr 17;9(4):e95246. doi: 10.1371/journal.pone.0095246 (PMC3990689; doi:10.1371/journal.pone.0095246)
Supplement: File S1 — Correspondence name nomenclatures and detailed structure descriptions. Table S1. Correspondence relationships between two nomenclatures of ABA receptors in Oryza sativa. Figure S1. The 2Fo-Fc electron density map of (+)-ABA and the interactive residues in OsPYL2. (+)-ABA was shown as yellow sticks. The red spheres stand for water, the blue stick for nitrogen atom and the red stick for oxygen atom. Figure S2. The pocket residues surrounding around (+)-ABA in OsPYL2 (left, green) and AtPYL2 (right, cyan). The conserved surrounding amino acids consist of the similar pocket size and topology between these two ABA receptors. The conserved residues are colored brown and (+)-ABA is colored yellow. (DOC) [file pone.0095246.s001.doc]

**Table S1 Correspondence relationships**

**between two nomenclatures of ABA receptors in *Oryza sativa***

| **Name** | **Gene**  **(RAP No.)** | **Gene**  **(MSU No.)** | **Gene**  **(NCBI No.)** | **Length (aa)** | **Correspondence**  **With Kim *et al.* study [12]** |
| --- | --- | --- | --- | --- | --- |
| **OsPYL1** | **Os10g0573400** | **LOC_Os10g42280.1** | **NP_001065470.1** | **212** | **OsPYL/RCAR10 (Os10g42280)** |
| **OsPYL2** | **Os06g0562200** | **LOC_Os06g36670.1** | **NP_001057874.1** | **207** | **OsPYL/RCAR9 (Os06g36670)** |
| **OsPYL3** | **Os02g0226801** | **LOC_Os02g13330.1** | **NP_001172865.1** | **211** | **OsPYL/RCAR2 (Os02g13330)** |
| **OsPYL4** | **Os01g0827800** | **LOC_Os01g61210.1** | **NP_001147241.1** | **257** | **OsPYL/RCAR1 (Os01g61210)** |
| **OsPYL5** | **Os05g0473000** | **LOC_Os05g39580.1** | **NP_001055819.1** | **216** | **OsPYL/RCAR6 (Os05g39580)** |
| **OsPYL6** | **Os03g0297600** | **LOC_Os03g18600.1** | **NP_001049838.1** | **229** | **OsPYL/RCAR4 (Os03g18600)** |
| **OsPYL7** | **Os06g0526400** | **LOC_Os06g33480.1** | **EEE65809.1** | **197** |  |
| **OsPYL8** | **Os06g0527800** | **LOC_Os06g33640.1**  **LOC_Os06g33640.2** | **NP_001057771.1** | **206** | **OsPYL/RCAR3 (Os02g15640)** |
| **OsPYL9** | **Os06g0528300** | **LOC_Os06g33690.1** | **NP_001057772.1** | **206** | **OsPYL/RCAR7**  **(Os06g33640)** |
| **OsPYL10** | **Os02g0255500** | **LOC_Os02g15640.1** | **NP_001046464.1** | **204** | **OsPYL/RCAR8 (Os06g33690)** |
| **OsPYL11** | **Os05g0213500** | **LOC_Os05g12260.1** | **NP_001054923.1** | **209** | **OsPYL/RCAR5 (Os05g12260)** |
| **OsPYL12** | **Os02g0255300** | **LOC_Os02g15620.1** | **BAD29692.1** | **181** |  |

**Figure S1**

**
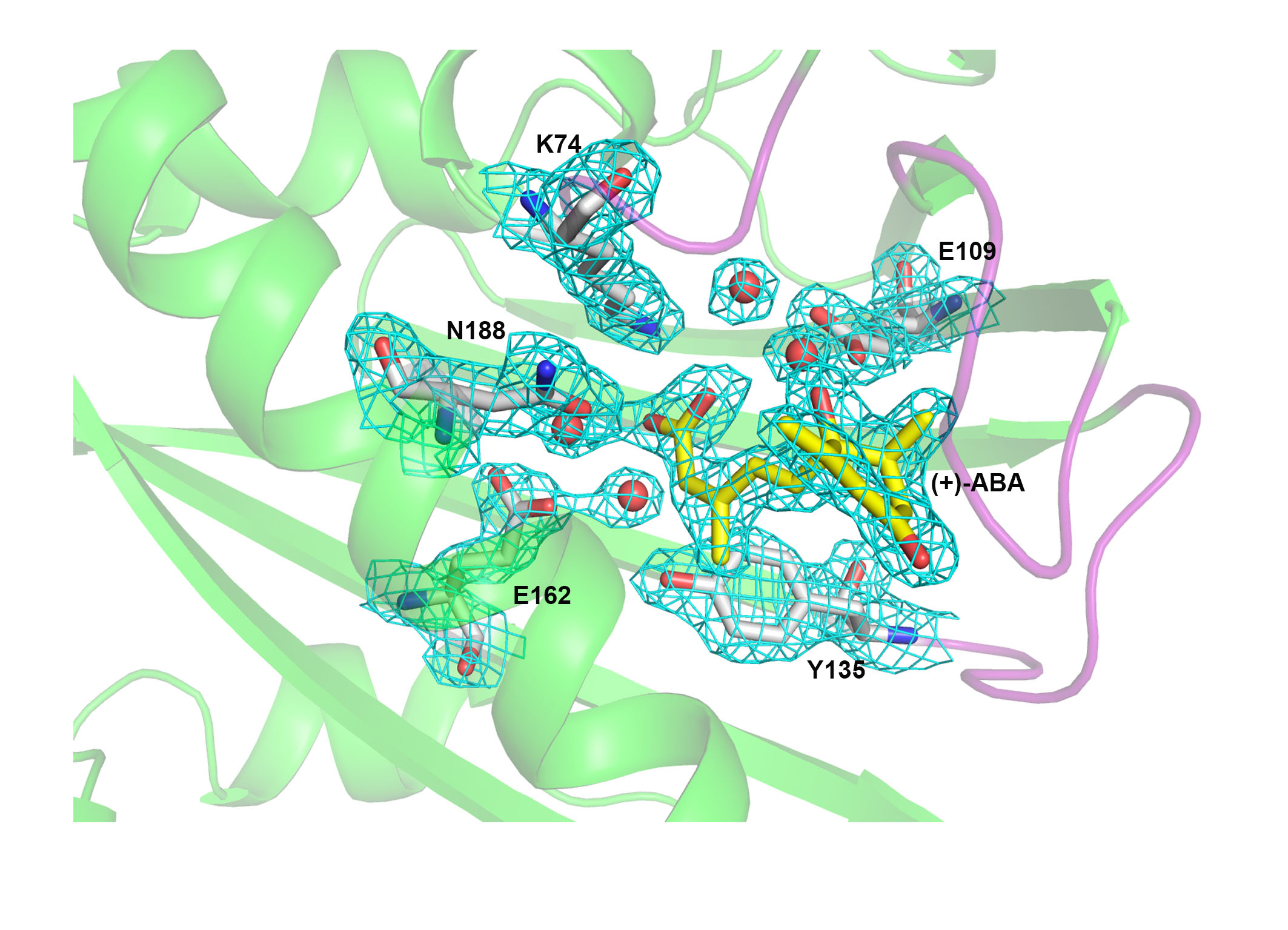
**

Figure S1. The 2Fo-Fc electron density map of (+)-ABA and the interactive residues in OsPYL2. (+)-ABA was shown as yellow sticks. The red spheres stand for water, the blue stick for nitrogen atom and the red stick for oxygen atom.

**Figure S2**


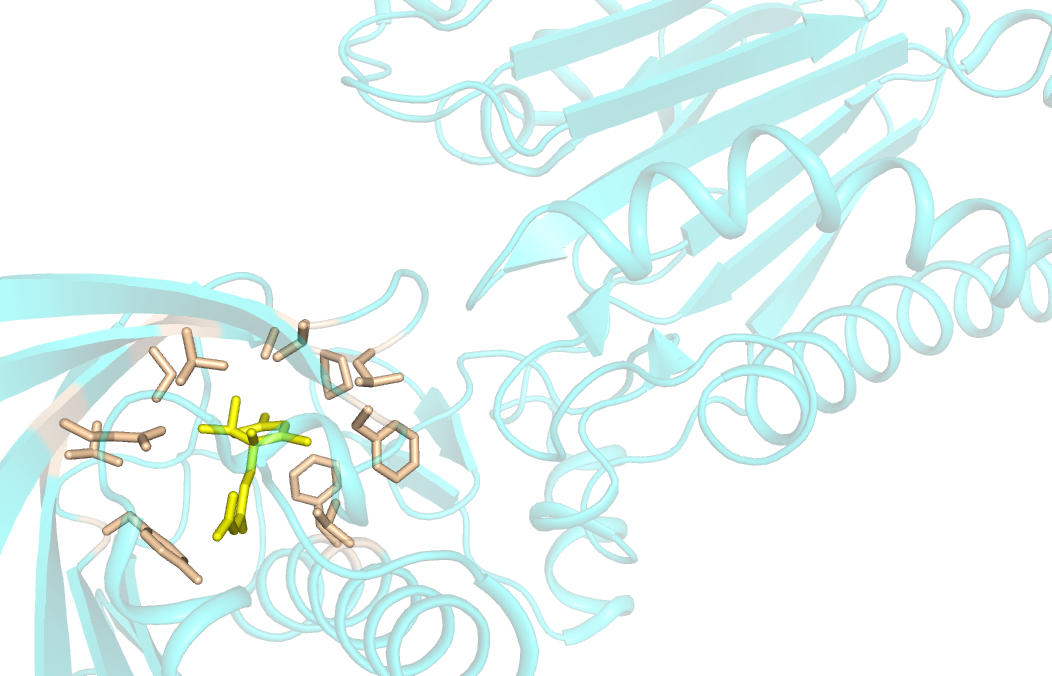


**Y124**

**V114**

**V86**

**S96**

**A93**

**V87**

**P92**

**L91**

**F66**

**V169**

**F165**

**E98**


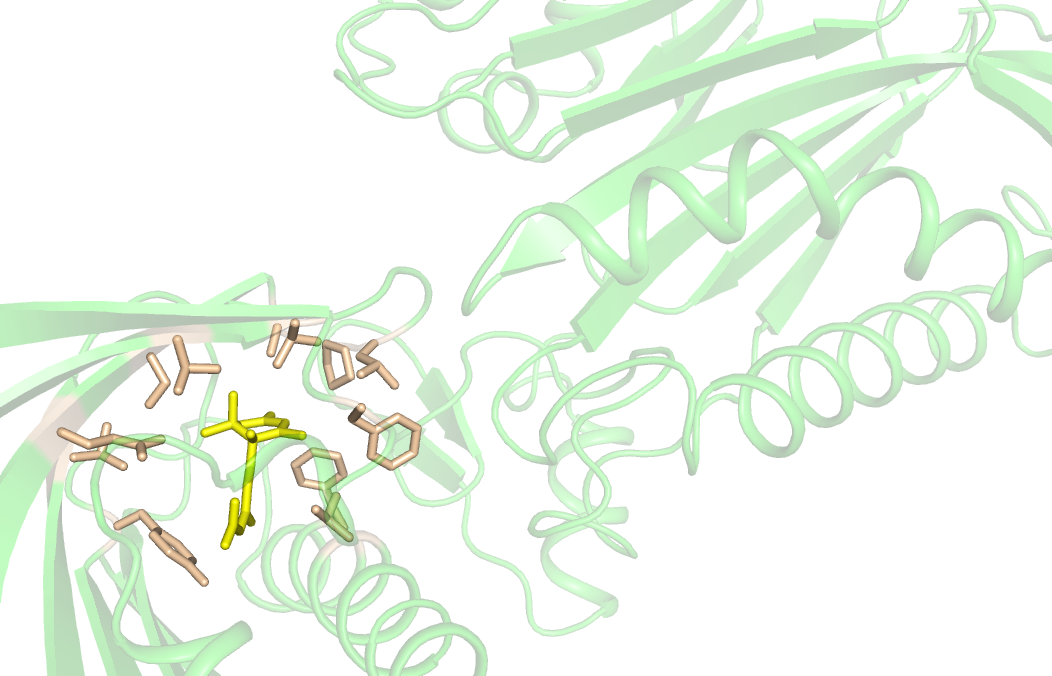


**V125**

**V98**

**L102**

**P103**

**A104**

**S107**

**V184**

**F76**

**F180**

**Y135**

**E109**

**V96**

Figure S2. The pocket residues surrounding around (+)-ABA in OsPYL2 (left, green) and AtPYL2 (right, cyan). The conserved surrounding amino acids consist of the similar pocket size and topology between these two ABA receptors. The conserved residues are colored brown and (+)-ABA is colored yellow.
